# Material and Substance Flow Analysis of Used Lead Acid Batteries in Nigeria: Implications for Recovery and Environmental Quality

**DOI:** 10.5696/2156-9614-10.27.200913

**Published:** 2020-08-25

**Authors:** Damilola Ogundele, Mary B. Ogundiran, Joshua O. Babayemi, Manis K. Jha

**Affiliations:** 1 Department of Chemistry, University of Ibadan, Ibadan, Nigeria; 2 Department of Chemical Sciences, Anchor University, Lagos, Nigeria; 3 National Metallurgical Laboratory, Jamshedpur, India

**Keywords:** lead acid battery, lead, motor vehicles, material and substance flow analysis, environmental quality

## Abstract

**Background.:**

As resources become scarce, information from material and substance flow analysis can help to improve material recovery policy. The flow of toxic substances such as lead (Pb), cadmium (Cd), chromium (Cr), arsenic (As) and antimony (Sb) can be used as a basis for appropriate risk management decisions for optimum environmental quality.

**Objectives.:**

The present study examined a material and substance flow analysis of used lead acid batteries (ULAB) from motor vehicles and implications for environmental quality in Nigeria.

**Methods.:**

Information on motor vehicle imports was obtained from the literature. Mathematical models were constructed and used for the material and substance flow analysis. Samples of 50 brands of ULAB pastes were digested using a microwave digestion system followed by elemental determination (Pb, Cd, silver (Ag), As, cobalt (Co), calcium (Ca), Cr, copper (Cu), iron (Fe), manganese (Mn), nickel (Ni), Sb, selenium (Se), and tellurium (Te)) with inductively coupled plasma optical emission spectroscopy.

**Results.:**

Approximately 4.8 million tons (Mt) lead acid batteries (LAB) from vehicles was used in Nigeria between 1980 and 2014, out of which approximately 2.6 Mt had reached end-of-life (EoL) stages. From the total amount in EoL, approximately 2.3 Mt was recycled, and 0.3 Mt was landfilled. Among the toxic elements, Pb, Cd and As were the most abundant in ULAB; and of the valuable elements, Fe and Cu had the highest levels. Approximately 3.5 Mt of Pb was used in the past (1980–2014) in ULAB for motor vehicles, out of which approximately 1.9 Mt tons was in EoL stages.

**Discussion.:**

The results revealed that the battery pastes were heterogeneous. Only Pb exceeded the total threshold limit concentration (TTLC) of 1000 mg/kg. The TTLC describes the safe levels or concentration of heavy metals in the environment. The levels observed for other metals in this study were below the TTLC values. The present study estimated an average life span for lead acid batteries in motor vehicles in Nigeria of 5 years, suggesting an additional 2.2 Mt at EoL by 2019. High concentrations of Pb in air, water and soil carry the potential for contamination of food products, especially in Nigeria, where food is traditionally prepared and sold in open air markets in an unregulated manner.

**Conclusions.:**

High amounts of toxic elements present in the various life cycle stages signal potential environmental and human health hazards.

**Competing Interests.:**

The authors declare no competing financial interests.

## Introduction

Chemical contamination through electrical and electronic material flow is of growing global concern as the fate of the chemical components of materials can threaten the environment and human health.^1^,^2^ Chemical contamination occurs when chemicals are introduced into the natural environment where their presence constitutes potential environmental and human health hazards. With reference to anthropogenic activities, chemical contaminants usually emanate from waste materials in various forms from either point or non-point sources.^3^ The effects are increasingly injurious around the world. As documented in Pure Earth, globally, over 200 million people are affected by toxic pollution; 8.7 million deaths occurred in low- and middle-income countries, out of the 9 million people killed by pollution worldwide in 2012.^4^ One major contributor to increasing chemical contamination is the increasing material and substance flow of electrical and electronic devices in circulation resulting from information and communication technological advancement and transportation.

When these devices reach end-of-life (EoL), they are recycled, landfilled or disposed of openly in the environment. In terms of transportation, when lead acid batteries reach EoL they may pose risks of chemical contamination if not managed in an environmentally sound manner.

One component of motor vehicles that is often replaced is the lead acid battery (LAB). The fate of the chemical components of this device at EoL may have a significant impact on the environment and human health if not well managed. A material/substance flow analysis of automobile used LAB (ULAB) is needed due to the volumes being generated and the concentrations of both valuable and toxic elements contained within ULAB. Material and substance flow analysis has often been preferred for the development of appropriate management plans, policy measures, estimates of exposure risks, understanding of low and high stocks for inventory, and dynamic forecasts.^5^–^8^ The detailed theory and practice of material and substance flow analysis is described in Babayemi *et al*.^8^

The aim of the present study was to evaluate the flow of automobile ULAB and their heavy metal content to understand the potential impacts on the environment in Nigeria. An estimate of ULAB generated in the country currently and in the future will help to better manage its flow. Nigeria has a large material flow of motor vehicles, and therefore a substance flow analysis of heavy metals in ULAB of motor vehicles in the country is needed.

## Methods

Material flow analysis is a technique applied to support material and substance flow management for e-waste mitigation. It is a method used for tracing the flow of a selected group of substances through a definite system. This type of substance flow analysis analyzes the availability of materials to justify the efficacy of recycling a product at its EoL. The data used for material flow analysis in the present study included the number of registered motor vehicles (cars, buses and trucks) imported into Nigeria between 1980 and 2014. Motor vehicles registration statistics in Nigeria from 1980 to 2014 were obtained from several national statistics and the literature.^9^ Data on the inflow of vehicles that carry LAB was obtained from Babayemi *et al*.^9^ Federal Road Safety Corps, national sources, Lagos State motor vehicle statistics, international trade statistics,^10^ as well as available reports and publications on the Nigerian transport sector. Relevant stakeholders (battery sellers, battery users and battery repairers) who were involved in the management and handling of ULAB were also consulted. The material flow analysis was conducted using STAN 2 software (inka software, 2012) from the Technical University of Vienna which assists in performing the analysis according to the Austrian Standard ONorm S 2096 (material flow analysis-application in waste management)^11^ in order to estimate the environmental/health impacts posed by improper management of ULABs. The software helped to build a graphical model with predefined components (processes, flows, system boundary, text fields), different layers (good, substance, energy) and periods to calculate unknown quantities. All flows can be displayed in such a way that the width of a flow is proportional to its value. The graphical picture of the model can be printed or exported.

**Abbreviations**
EoL: End-of-life
LAB: Lead acid batteries
Mt: Million tons
TTLC: Total threshold limit concentration
ULABs: Used lead acid batteries

### Lead acid batteries types and management in Nigeria

A research survey was designed to obtain information on the available LAB types in Nigeria, their life span and EoL management. A survey of automobile LAB types was carried out in markets in southwestern Nigeria (i.e. in Lagos, Ogun, Oyo, Osun, Ondo, Ekiti and Kwara States) *(Supplemental Material).* In each of these states, 200 questionnaires were distributed for a total of 1,400. However, in one of the states, only 100 questionnaires were completely filled out and returned by the respondents. One hundred (100) completely filled-out questionnaires were also selected from each of the other states, for a total of 700 completely filled-out questionnaires retrieved from the respondents. The respondents included ULAB sellers, users, repairers, and scavengers.

### Import of motor vehicles and life cycle stages

Information on the number of motor vehicles imported, those in current use and those that reached EoL between 1980 and 2010, were obtained from Babayemi *et al*.^9^ Vehicles in current use were comprised of all registered vehicles that were still on the road up to 2010. Data for motor vehicles imported in 2011–2014 were obtained from UN Comtrade database.^10^ This information from the literature and database were used for further calculations, using Equation 1, for simple estimation (the equations are algebraic approaches to make estimations simpler). The approach has been effectively and successfully applied in other studies.^12^,^13^

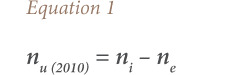
where, n_i_ is the number of motor vehicles imported (1980–2010); n_e_ is the number in EoL; and n_u (2010)_ is the number in use up to 2010. It was assumed that the motor vehicles imported from 2010 till 2014 were still in current use in 2014.

### Lead-acid battery components of motor vehicles in Nigeria

The average life span of motor vehicles that were in EoL in 2004 in Nigeria was 25 years (1980–2004). Based on this information, the number of ULAB in EoL between 1980 and 2004 was estimated; and all other vehicles between 2011 and 2014 were assumed to still be in current use. From these totals, the total amount of batteries used by motor vehicles in Nigeria (1980–2014) was calculated using the relationships in Equations 2–4. This was done in two steps: one, for motor vehicles that reached EoL within this period of time; and two, for those in use up to the inventory year 2014, using the same relationships.

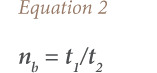


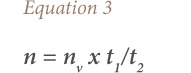


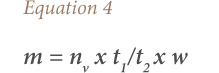
where, *n_b_* is the number of batteries used per vehicle; *t_1_* and *t_2_* are the life spans of a vehicle and battery, respectively (the average life span of batteries was taken to be 5 years based on the survey in this study); *n* is the total number of batteries used by a category of vehicle; *n_v_* is the number of vehicles in a category; w is the average weight (tons) of battery (the average weights of LAB for cars, buses and trucks were obtained from the literature (America Public Transportation Association)); and *m* is the total amount (tons) of batteries used by a category of vehicle.^14^

However, the amounts of motor vehicles imported to Nigeria between 2011 and 2014 were reported in weights in the UN Comtrade database (not in absolute numbers). The average percentage of LAB per vehicle (the amount of battery) was calculated.

### Collection of end-of-life lead acid batteries and pre-treatment

A total of 50 different brands of ULAB were randomly collected in Ilorin, Kwara State and Ado Ekiti in Ekiti State, based on the similarity of type revealed by the survey. The most common ULAB observed in the market was a calcium (Ca) alloy ULAB. The samples were collected from individual users and scavengers based on the type and brand. These samples were transported to the laboratory prior to analysis. They were dismantled individually into different components. Prior to dismantling, they were discharged to ensure neutrality of the terminals.

The pastes, being the major source of heavy metals, were separated from the remaining battery components. The pastes were slurred in 70°C water for 1 hour on a magnetic stirrer operated at 450 rpm on a hot plate to determine neutrality. They were dried, homogenized and characterised for elemental composition.

### Elemental characterization of lead acid battery pastes

The grids and the paste were not separated but processed together. Of each of the pastes, 0.20 g was digested with 10 mL each of nitric acid, hydrochloric acid and perchloric acid concentrations using Anton Paar's Multiwave 3000 microwave digestion system. After dissolution, the digested samples were filtered into a 250 mL standard flask and made up to the mark with distilled water. Lead (Pb), Ca, cadmium (Cd), silver (Ag), arsenic (As), cobalt (Co), chromium (Cr), copper (Cu), iron (Fe), manganese (Mn), nickel (Ni), antimony (Sb), selenium (Se), and tellurium (Te) were determined in the digest with inductively coupled plasma optical emission spectrophotometer (iCAP 7600 ICP-OES, Thermo Scientific, Austria).

### Elements in the spent lead acid batteries

The amounts of elements in the ULAB were estimated using Equation 5.

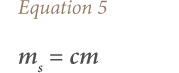
where, m_s_ indicates the amount of elements in the spent lead acid battery; *c* is the concentration (in percentage) of each element in the battery; and *m* is the total amount (tons) of battery used by a category of vehicle in a particular life cycle stage.

## Results

The survey revealed that about 98% of automobile LABs in Nigeria were Ca ion batteries, while the remaining 2% were magnesium ion batteries. From the results of active participants in the management of ULABs in Nigeria, 11% of the respondents identified themselves as battery sellers, 17% as battery repairers, 23% as scavenger/recyclers and 49% as battery users. Assessment of the life span of LAB as indicated by users showed that 25% of the respondents used batteries for 3 years, 59% for 5 years and 16% for 7 years.

**Figure 1:**
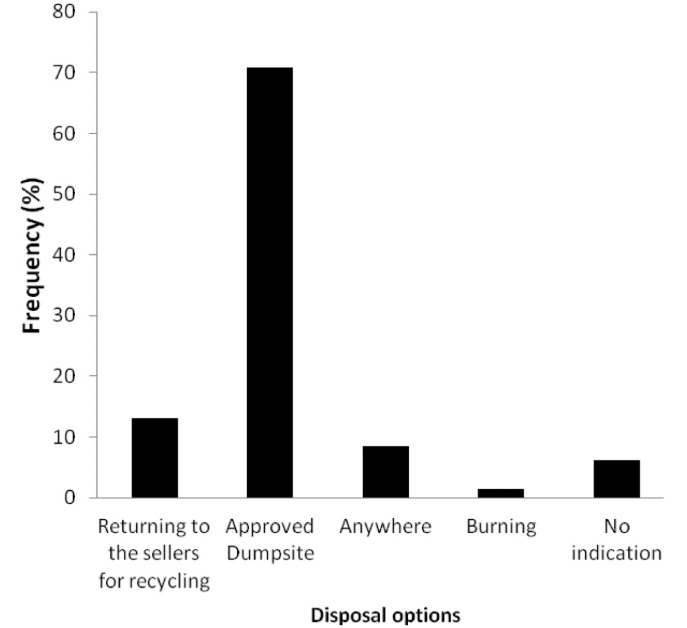
Current disposal options for spent lead acid batteries in Nigeria

The results of EoL management of ULABs among participants are shown in Figure 1. About 8.5% of the respondents disposed of ULABs indiscriminately, 70.8% in officially approved dumpsites, 1.5% burnt the spent products, 13.1% returned the used battery to sellers and 6.2% could not account for the management of used products.

### Material flow of automobile lead acid batteries in Nigeria (1980–2014)

The results of the registered number of vehicles imported to Nigeria from 1980 to 2010 are presented in Table 1. Approximately 19 million motor vehicles (cars, buses and trucks) were imported to Nigeria. Of these, about 2.4 million cars, 984,725 buses and 34,225 trucks were estimated to be at their EoL by 2004. Over 10 million cars, 5 million buses and about 160 thousand trucks were in use from the estimated vehicles reported to have entered Nigeria between 1980 and 2010, spanning a period of 30 years.^9^

**Table 1:** Numbers of Vehicles Registered in Nigeria from 1980 to 2010

**Vehicle**	**Number imported (1980–2010)^9^**	**Number in EoL up to 2004^9^**	**Number in use up to 2010**
Cars	13,000,000	2,395,912	10,604,088
Buses	6,000,000	984,725	5,015,275
Trucks	190,000	34,225	155,775
Total	19,190,000	3,414,862	15,775,138

The lifespan of LAB for vehicles in Nigeria is 5 years, dividing the lifespan of a motor vehicle by 5 will give the number of LAB used by the vehicle in its lifetime. As presented in Table 2, approximately 3 million motor vehicles in EoL up to the year 2004 used approximately 390,000 tons of LAB. Motor vehicles imported in 1980 and in use up to 2010 would each have used up to six (6) LABs. Thus, the approximately 16 million motor vehicles in use up to the year 2010 would have used an estimated 2 million tons (Mt) of LAB *(Table 3).*

**Table 2:** Estimated Distributions of Lead Acid Batteries at End-of-Life up to 2004

**Vehicle**	**Number in EoL up to 2004**	**Battery lifespan (year)**	**Number of batteries used per vehicle**	**Total Number of batteries used**	**Average weight of battery (kg)^14^’^15^**	**Total amount of battery (tons)**
Cars	2,395,912	5	5	1,1979,560	22.7	271,936
Buses	984,725	5	5	4,923,625	22.7	111,766
Trucks	34,225	5	5	171,125	24	4,107
Total	3,414,862					387,809

**Table 3:** Estimated Distributions of Lead Acid Batteries in Motor Vehicles in Use up to 2010

**Vehicle**	**Number in use up to 2010 (30 years)^9^**	**Battery lifespan (yr)**	**Number of batteries used per vehicle**	**Total Number of batteries used**	**Average weight (kg) of battery**	**Total amount of battery (tons)**
Cars	10,604,088	5	6	63,624,528	22.7	1,444,277
Buses	5,015,275	5	6	30,091,650	22.7	683,080
Trucks	155,775	5	6	934,650	24	22,432
Total	15,775,138					2,149,789

Import data for motor vehicles in Nigeria between the year 2011 and 2014 were obtained from the UN Comtrade database *(Table 4).* However, the amount was reported in mass (kg). The approximately 6.7 Mt of motor vehicles imported within this period of time used approximately 54,000 tons of LAB *(Table 5).* Altogether, approximately 4.8 Mt of LAB was used over thirty-four years (1980–2014) in the country *(Table 6).*

**Table 4:** Amount (kg) of Buses, Cars and Trucks Imported to Nigeria (2011–2014)

**Period**	**Net weight (kg)**			

	**Buses**	**Cars**	**Trucks**	Total
2011	1,114,913,120	1,526,360,204	552,604,406	
2012	343,081,497	1,550,482,497	527,426,823	
2013	85,147,701	583,364,153	228,836,281	
2014	33,599,107	112,080,943	84,856,638	
Total	1,576,741,425	3,772,287,797	1,393,724,148	6,742,753,370

**Table 5:** Amount (kg) of Lead Acid Batteries Imported via Buses, Cars and Trucks into Nigeria (2011–2014)

**Vehicle**	**Amount**	**Fraction of LAB (%)^14^**	**Amount of battery**
Buses	1,576,741,425	0.25	3,941,854
Cars	3,772,287,797	1.25	47,153,597
Trucks	1,393,724,148	0.20	2,787,448
Total	6,742,753,370		53,882,899

**Table 6:** Total Amount (tons) of Lead Acid Battery Used via Buses, Cars and Trucks in Nigeria (1980–2014)

**Vehicle**	**Amount in EoL vehicles (1980–2010)**	**Amount in use up to 2010**	**Amount in use(2011–2014)**	**Amount in vehicles in current use (2014)**	**Total amount used in history (1980–2014)**
Cars	271,936	1,444,277	47,154	1,491,431	3,254,798
Buses	111,766	683,080	3,942	687,022	1,485,810
Trucks	4,107	22,432	2,788	25,220	54,547
Total	387,809	2,149,789	53,884	2,203,673	4,795,155

### Elemental composition of end-of-life lead acid battery pastes

The elemental compositions and concentrations of EoL automobile ULAB pastes in Nigeria are shown in Table 7. The results revealed that the battery pastes were heterogeneous. Only Pb exceeded the total threshold limit concentration (TTLC) of 1,000 mg/kg. The percentage weight of Pb in the paste was 72.1% (721,000 ± 38.2 mg/kg).

**Table 7:** Average Concentration of Heavy Metals (mg/kg) in Lead Acid Batteries and Total Threshold Limit Concentration Values

**Heavy Metals**	**Samples Mean ± S.E (mg/kg)**	TTLC (mg/kg) and ^*^μg/kg
Pb	721,000 ±38.2	1000
Cd	7.90 ±7.0	100
Ag	13.4 ±7.4	500
As	6.00 ±3.0	500
Co	10.00 ±6.0	8000
Ca	89.00 ± 0.2	-
Cr	1.10 ±0.8	2,500
Cu	310±255.8	2,500
Fe	850 ±248.0	1000
Mn	54.0 ±38.0	2,500
Ni	< nd	2000
Sb	170 ±42.0	500
Se	0.6 ±0.3	100
Te	1.40 ±0.5	^*^100

^*^Total threshold limit concentration by California Department of Toxic substances control. Code of regulation: 22 sections 66261.24.^16^

### Material flow of motor vehicles in Nigeria (1980–2014)

Using an average weight for a car of 1,250 kg; bus as 1,850 kg; and truck as 2,649 kg, for the years 2011–2014, approximately 3,017,830 cars, 852,293 buses and 526,132 trucks were imported into Nigeria.^13^ Babayemi *et al*. previously reported that approximately 19 million motor vehicles were imported to Nigeria between the year 1980 and 2010; which suggests that approximately 23 million motor vehicles were imported to the country in the years 1980–2014.^9^

The inflow of vehicles into Nigeria has drastically increased within the past few decades. Eighty-five percent (85%) of vehicles imported into Nigeria are second-hand vehicles aged between 5 to 15 years, in spite of the prohibition of vehicles older than 7 years entering into Nigeria.^17^

### Material flow of lead acid batteries of motor vehicles in Nigeria (1980– 2014)

**Figure 2:**
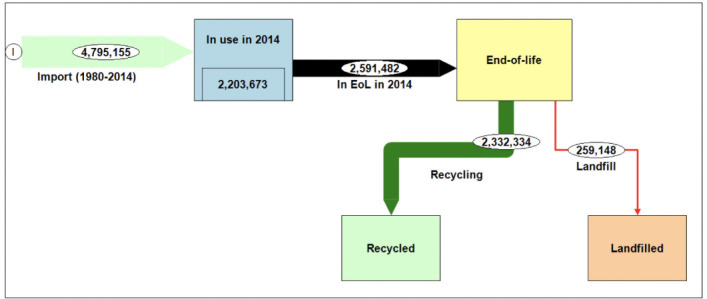
Material flow chart of lead acid batteries of motor vehicles imported into Nigeria (1980–2014), stock (tons) for inventory year 2014

Between 1980 and 2014 *(Figure 2),* approximately 4.8 Mt of LAB were used via motor vehicles in Nigeria; of which approximately 2.6 Mt have reached EoL; while approximately 2.2 Mt were in current use in 2014.

### Substance flow analysis of valuable and toxic elements in automobile lead acid batteries in Nigeria

The results of the elemental flow in automobile LABs are shown in Table 8. Approximately 3.5 Mt of Pb were used from 1980–2014 via LAB from motor vehicles in Nigeria, of which approximately 1.9 Mt were at EoL, and approximately 1.6 Mt in current use in the year 2014. Cadmium is another toxic element found in LAB. The substance flow of this element revealed that 37.8 tons were used between 1980 and 2014; 20.4 tons contained in LAB in EoL and 17.4 tons in current use. The amount of As used from 1980–2014 via LAB was 28.7 tons; while 15.5 tons were contained in LAB at EoL and 13.2 tons in current use. Substance flow analysis of valuable elements like Fe, Cu and Sb revealed considerable amounts of these elements. Approximately 4,000 tons of Fe has been used from 1980–2014 via LAB, 2,200 tons in EoL and approximately 2,000 tons in current use. Approximately 1,500 tons of Cu was used from 1980–2014, 800 tons in EoL, and 680 tons in current use. Considerable amounts of Sb were also used from 1980–2014, approximately 800 tons, with approximately 440 tons contained in EoL LAB, and 400 tons in current use. High levels of these valuable elements indicate good potential for their quantitative recovery.

**Table 8:** Substance Flow Analysis of Valuable and Toxic Elements in Lead Acid Batteries

**Heavy Metals**	**Amount (tons) in batteries of motor vehicles (1980–2014)**			**Amount (tons) in different life cycle stages**		
	**Cars**	**Buses**	**Trucks**	**Total amount in history (1980–2014)**	**Amount in end-of-life (1980–2014)**	**Amount in current use (2014)**
Pb	2,346,709	1,071,269	39,328	3,457,306	1,868,458	1,588,848
Cd	25.7	11.7	0.4	37.8	20.4	17.4
Ag	43.6	20.0	0.7	64.3	34.8	29.5
As	19.5	8.9	0.3	28.7	15.5	13.2
Co	32.5	14.9	0.5	47.9	25.9	22
Ca	290	132	4.9	427	231	196
Cr	3.58	1.6	0.06	5.24	2.84	2.4
Cu	1,009	461	17	1,487	804	683
Fe	2,767	1,263	46	4,076	2,203	1,873
Mn	176	80	3	259	140	119
Sb	553	253	9.3	815	440	375
Se	2	1	0.03	3.03	1.73	1.3
Te	4.6	2.1	0.08	6.78	3.68	3.1

However, Cd is not in common use in LAB anymore, and Sb is being replaced by Ca. It is not likely to be profitable to recover Cd, because there is a shrinking market for the metal due to environmental concerns. Antimony is much easier to recover, but with LAB manufacturers moving to Ca alloys for the grids, the market for Sb is also shrinking.

## Discussion

The results of LAB and EoL management in Nigeria imply that the largest fraction of ULAB end up in dumpsites. This trend is similar to the disposal option for batteries from waste rechargeable lamps in Nigeria of which about 69% end up in dumpsites.^18^ However, Osibanjo and Suleiman found that 90% were recycled.^19^,^20^ It was reported that about 90% of ULAB are ultimately recycled for lead; that is, amongst other components of the battery. This indicates that Pb is the component most desired by battery recyclers. In Nigeria, there are few organized recyclers; the majority are repairers and small-scale smelters. The present study sought to determine the percentages of ULAB which were returned to the formal recycling system, disposed of indiscriminately, burnt, or transported to approved dumpsites where scavengers pick them up, or which remained in the dumpsite. A total of 13.1% were returned to sellers. Many users were unaware of this possibility, and thus discarded their used batteries. The reports of Osibanjo and Suleiman describe the percentage of components recycled, such as Pb, in ULAB in EoL, rather than 90% of the entire ULAB; otherwise, it might be wrongly assumed that Nigeria has a highly efficient formal recycling system of ULAB. In Nigeria, ULAB are unlikely to remain in the dump, because scavengers and others will remove them, as they have a commercial value.

The elemental (Pb) compositions and concentrations of ULAB pastes observed in this study are similar to the concentration range of 70–73% reported by Buzatu *et al*.^21^ The levels observed for metals other than Pb in this study were below the TTLC values. It should be noted that the LAB samples in the present study were randomly collected and not separated based on vehicle source (cars, buses or trucks) or manufacturer. The material and substance flow analysis based on cars, buses and trucks assumed similar percentage elemental battery compositions.

With reference to the observations of Suleiman and Osibanjo that about 90% of LAB in EoL were recycled and 10% were landfilled,^19^,^20^ this implies that, of the amount of ULAB in EoL, approximately 2.3 Mt were recycled and 0.3 Mt were landfilled. Given an average life span of LAB of motor vehicles in Nigeria of 5 years as estimated in the present survey, by the year 2019, approximately 2.2 Mt was added to the amount in EoL. Appropriate policy and infrastructural frameworks should be put in place to handle this large amount of LAB waste.

### Implications for environmental quality in Nigeria

An ecological risk assessment of heavy metal pollution of the agricultural ecosystem near a LAB factory found that the contamination factor values of Pb ranged from 2.8–5.3; which indicates that the examined soils were strongly impacted by Pb.^22^ Increased anthropogenic activity is a significant contributor to global occurrences of Pb in the environment, including in air, water, soil, food and humans. Elevated levels may now be observed even in the most natural environments.

Higher levels of Pb have been reported in surface water in Nigeria, exceeding water quality criteria for aquatic life.^23^–^25^ Lead levels in these surface water bodies may pose both acute and chronic toxicity to the aquatic organisms, thereby putting the ecosystem at risk. Furthermore, significant numbers of the Nigerian population, especially rural dwellers, depends on surface water for direct use for domestic activities, including cooking.^3^

The challenge of water contamination by Pb is not limited to surface water only; higher levels have also been reported for groundwater sources in Nigeria.^26^–^28^ Groundwater may then constitute a major medium of human long-term exposure to Pb in the country.

Various levels of Pb have been reported for soils from automobile workshops in the country, ranging from 0.15–15,100 mg/kg.^29^–^35^ Lead soil quality guidelines for human health are 140 mg/kg for agricultural land and residential/park land, 260 mg/kg for commercial land use, and 740 mg/kg for industrial land.^36^ Lead levels in soil from automobiles were much higher, with factors above 100, and reached extremely hazardous levels. 26–32

High levels of Pb in atmospheric particulates or deposited dusts have also been reported in Nigeria, exceeding the 0.5 μg/m^3^ European Union Air Quality Standard for Pb, implying risks to human health.^37^–^40^ Sources of Pb in air include automotive and battery recycling.^37^,^41^ In addition, high levels of Pb have been measured in indoor air and household dust.^42^ A significant relationship was found between levels of Pb in dust and in the blood of children in public schools.^43^

Although not associated with LAB or ULAB activities, several deaths were connected to Pb poisoning caused by artisan methods of metallurgical extraction used by gold miners in Zamfara State, northeastern Nigeria in 2010. 44 Over 10,000 people were estimated to have been affected, and over 700 children were reported to have died as a result from 2010 to 2013. In addition, 28 children were reported to have died in May 2015 in Niger State due to Pb poisoning.^44^ In light of the scope of Pb contamination of the Nigerian environment, most likely a significant proportion of the population may carry Pb in their tissues. Previous studies have reported that occupationally exposed auto mechanics in Abeokuta, Ogun State had a Pb level of 14.3±5.90 mg/kg (scalp hair) and 5.31±2.77 mg/kg (nail); and urine samples in Bauchi contained 0.01–0.14 mg/L.^45^,^46^ The main sources of human exposure to heavy metals in Nigeria come from contamination of air, water, soil and foods.^3^ Contact with playground soil and indoor classroom dust particles are likely sources for children.^42^,^43^ Occupational behavior predisposes many informal sector workers to heavy metals. For instance, many of those involved as auto mechanics do not use personal protective equipment, and many have been in the business for over ten years.^47^

Finally, numerous battery chemicals such as acid electrolytes are corrosive, and when released into the environment pose hazards and harm plants and animals. In addition, since the recycling process is carried out manually by the informal sector, skin contact poses a risk to human health. However, acid electrolytes of samples used in the present study were checked and pH was found to be close to neutral, as water and Pb(II) sulfate dominated the solution after it was spent or at EoL.

### Study limitations

In each of the states (Lagos, Ogun, Oyo, Osun, Ondo, Ekiti and Kwara States), 200 questionnaires were distributed for a total of 1,400. However, in one of the states, only 100 questionnaires were completely filled out and returned by the respondents. This can result in bias. Furthermore, details of uncertainties associated with UN Comtrade database has been reported.^48^

## Conclusions

The present study investigated the material and substance flow analysis of ULAB to visualize amounts in different life cycle stages and determine implications for environmental quality in Nigeria. High amounts of toxic elements present in the various life cycle stages signal potential environmental and human health hazards.

The current investigation of material and substance flow analysis of ULAB in Nigeria will provide a better understanding of this material and help to evolve waste management policies and/or recycling schemes which will be the focus of future studies. Aside from Pb, the flow of other toxic substances such as Cd, Cr As and Sb was also studied, as a basis for appropriate risk management decisions. This study can lead to or form a basis of life cycle assessment of LABs. Furthermore, as resources become scarce, information from material and substance flow analysis can help to improve material recovery policy, as demonstrated by the flow of valuable substances such as Ag, Co, Cu, Fe, Mn, Ni, Se, and Te. The present study also demonstrated the need for an appropriate recycling strategy for ULABs. This becomes imperative in a circular flow economy.^49^,^8^

There is need for a safe environmental and green approach to the management of ULABs. Recovery of Pb from ULABs has reached advanced stages in developed countries but is still very rudimentary in developing countries. The most widely available method in developing countries is pyrometallurgical.^13^ The pyrometallurgical system involves the smelting of battery paste at high temperature to recover valuable materials. This method is used by most legal refining plants and has also been abused by local illegal refiners. This poses environmental and health risks to the ecosystem. The hydrometallurgical method on the other hand involves the use of chemicals for the recovery of valuable metals at much lower temperatures than the pyrometallurgical method. It is therefore recommended that regulators and manufacturers of LAB establish an environmentally friendly way of recovering Pb from ULABs.

Finally, the high volume of Pb in various life cycle stages and possible contamination of related media may result in human exposure. There is a need for scientific and legal actions for remediation where necessary to prevent or reduce further pollution. In particular, there is an urgent need for carrying out appropriate sustainable waste management and pollution prevention strategies which include (i) reduction of waste (ii) recycling of waste (iii) reuse of waste (iv) re-design/re-manufacturing of products (v) remediation of contaminated soil, and (vi) research and monitoring.^3^
