# Supplementary material for: Material and Substance Flow Analysis of Used Lead Acid Batteries in Nigeria: Implications for Recovery and Environmental Quality
Source: J Health Pollut. 2020 Aug 25;10(27):200913. doi: 10.5696/2156-9614-10.27.200913 (PMC7453816; doi:10.5696/2156-9614-10.27.200913)
Supplement: Supplementary file 1 [file Babayemi_Supplemental_Material.pdf]

Appendix X: Research questionnaire

Survey Questionnaire

Section A: Personal Data
Please tick as appropriate:

Sex: Male [1] Female [2] Age: below 20 [1] above 30[2]

Occupation: Battery Importers/Sellers [1] Battery Repairers [2]

Battery User [3] Local Recyclers (waste scavenging) [4]

Section B:
Please tick as appropriate

D = Disagree/No (1) AG = Agree/Yes (2)

S/N	Questions	Disagree<br>No	Agree<br>Yes	Others (specify)
1	Do you know Pb-acid battery?	1	2	
2	How many Pb-acid battery have you used?	1	2	
3	What brand/types of lead acid battery do you like/use?	1	2	
4	Do you receive used LAB and how many do you receive from customer in a year............?	1	2	
5	How long do the battery last? Specify years	1	2	
6	Do you know that People engage in manual recycling of LABs	1	2	
7	What are they looking for in the battery? a. lead Pb b. Acids c. Plastic	1	2	
8.	Are you aware of Spent Lead acid battery recycling facilities/company in Nigeria	1	2	
9	Do you have a Car/Motor cycle/Bus/ mini bus(Akoto) that use lead acid battery	1	2	
10	There is market for used battery waste, thus scavenging is lucrative	1	2	
11	Do you know that foreign countries where these used batteries are being imported prohibit the burning or manual recycling?	1	2	
12	Have you ever repaired your battery?	1	2	
13	How many times have you changed your car battery? a. once b. twice c. thrice	1	2	

14	Have you ever left your used battery with battery chargers	1	2	
15	Have you dumped your used battery in the waste bin before	1	2	
16	Do you have used battery in your surrounding or Garage	1	2	
17	Have you at one time or the other dumped battery in dumpsites	1	2	
